# The Impact of Sleep Restriction and Simulated Physical Firefighting Work on Acute Inflammatory Stress Responses

**DOI:** 10.1371/journal.pone.0138128

**Published:** 2015-09-17

**Authors:** Alexander Wolkow, Sally A. Ferguson, Grace E. Vincent, Brianna Larsen, Brad Aisbett, Luana C. Main

**Affiliations:** 1 Centre for Physical Activity and Nutrition Research, Deakin University, 221 Burwood Hwy, Burwood, VIC 3125, Australia; 2 Bushfire Co-Operative Research Centre, East Melbourne 3002, Australia; 3 Central Queensland University, Appleton Institute, Wayville 5034, Australia; Academic Medical Centre/University of Amsterdam, NETHERLANDS

## Abstract

**Objectives:**

This study investigated the effect restricted sleep has on wildland firefighters’ acute cytokine levels during 3 days and 2 nights of simulated physical wildfire suppression work.

**Methods:**

Firefighters completed multiple days of physical firefighting work separated by either an 8-h (Control condition; n = 18) or 4-h (Sleep restriction condition; n = 17) sleep opportunity each night. Blood samples were collected 4 times a day (i.e., 06:15, 11:30, 18:15, 21:30) from which plasma cytokine levels (IL-6, IL-8, IL-1β, TNF-α, IL-4, IL-10) were measured.

**Results:**

The primary findings for cytokine levels revealed a fixed effect for condition that showed higher IL-8 levels among firefighters who received an 8-h sleep each night. An interaction effect demonstrated differing increases in IL-6 over successive days of work for the SR and CON conditions. Fixed effects for time indicated that IL-6 and IL-4 levels increased, while IL-1β, TNF-α and IL-8 levels decreased. There were no significant effects for IL-10 observed.

**Conclusion:**

Findings demonstrate increased IL-8 levels among firefighters who received an 8-h sleep when compared to those who had a restricted 4-h sleep. Firefighters’ IL-6 levels increased in both conditions which may indicate that a 4-h sleep restriction duration and/or period (i.e., 2 nights) was not a significant enough stressor to affect this cytokine. Considering the immunomodulatory properties of IL-6 and IL-4 that inhibit pro-inflammatory cytokines, the rise in IL-6 and IL-4, independent of increases in IL-1β and TNF-α, could indicate a non-damaging response to the stress of simulated physical firefighting work. However, given the link between chronically elevated cytokine levels and several diseases, further research is needed to determine if firefighters’ IL-8 and IL-6 levels are elevated following repeated firefighting deployments across a fire season and over multiple fire seasons.

## Introduction

Each year, firefighters are deployed to combat the threat of large wildfires to property and lives. These deployments can last multiple days and require firefighters to perform extended hours (i.e., 12 to 15 h) of intense, intermittent, physical work with restricted sleep opportunities between shifts (i.e., 3 to 6 h) [1, 2, 3]. Evidence suggests that individually, physical work [4] and sleep restriction [5–7] can elicit an acute inflammatory response causing the release of cytokines.

Pro-inflammatory cytokines such as interleukin (IL)-1β, Tumour Necrosis Factor (TNF)-α and IL-8 facilitate an acute-phase response [8–10]. Conversely, anti-inflammatory cytokines such as IL-10 inhibit pro-inflammatory cytokines and attenuate inflammation [9, 11]. Furthermore, IL-6 and IL-4 cytokines display both pro- and anti-inflammatory activities that modulate inflammation [12–15]. Together, these processes coordinate the body's acute inflammatory response to a stressor to maintain homeostasis of the immune system. However, severe or chronic stress exposure may exacerbate the immune response resulting in chronically elevated cytokine levels and associated adverse health outcomes [9, 16].

Acute increases in IL-6 [7] and TNF-α [5, 6] have been observed after 5–7 nights of sleep restricted to 4 h or 6 h per night in the laboratory, without physical work. Chronically elevated TNF-α and IL-6 levels are markers of systematic inflammation linked to negative health outcomes such cardiovascular disease (CVD) and insulin resistance [17, 18]. Increased IL-6 and IL-8 levels were also reported following 3-days of intense physical running training (2.5 h/day) without sleep restriction [19]. Chronically elevated IL-8 levels are also associated with atherogenesis and inflammatory changes that may result in CVD [20]. In a field setting, Main et al. [4] reported increased IL-6 across a shift of physical wildfire work without sleep disruption. However, firefighters’ IL-6 levels, along with IL-1β, IL-8 and IL-4 all exhibited an attenuated response across the second shift, possibly indicative of an adaptation [4].

While firefighting literature is sparse, multi-day military and exercise-based studies have reported an increase [21], decrease [22] or fluctuation in IL-6 [23, 24]. Increased, unchanged or fluctuations in IL-1β, TNF-α and IL-10 levels were also reported among soldiers completing seven consecutive days of physical work with minimal sleep (e.g., 7 h total) [23, 24]. Though it is possible the inflammatory markers in these field-based studies were confounded by other stressors (e.g., fluid and energy intake), an attenuated or unchanged cytokine response to these demands may indicate a non-damaging regulatory response. For instance, the immunomodulatory properties of IL-6 modulate pro-inflammatory cytokines [12, 13, 25, 26] that underpin systemic inflammation [27, 28]. The immune system also interacts with cortisol [29], found to increase during simulated wildland firefighting work [30]. An acute increase in cortisol can down-regulate cytokine activity to maintain homeostasis of the immune system [29, 31, 32]. While military- and exercise-based research provide some understanding of the effect of physical work and sleep loss on cytokine responses, the demands investigated differ to the sleep restriction and physical work involved in wildfire suppression. Extrapolation of findings to wildland firefighting could, therefore, under- or over-estimate any stress-related implications.

Military-based research mostly investigated long duration marching and running [22–24], whereas wildland firefighting work incorporates a large component of short-duration weight bearing manual handling tasks, in addition to sustained aerobic activity [33]. Given that eccentric contractions are known to produce a more pronounced increase of IL-6 and IL-8 compared to concentric contractions [27], military-based findings could lead to under-estimates of the cytokine response for wildfire personnel. Furthermore, total sleep deprivation is associated with a greater elevation in IL-6 than partial sleep restriction [34]. The almost complete sleep restriction examined in military-based studies [23, 24] could over-estimate the cytokine response for firefighters who report 3 to 6 h of sleep per night [1]. Under- or over-estimating inflammation could lead to inappropriate recommendations regarding the management of risk associated with firefighter’s sleep during deployments. The aim of the present study was therefore, to assess the effect restricted sleep has on wildland firefighters’ inflammatory cytokine levels during 3 days and 2 nights of simulated physical firefighting work.

## Materials and Methods

### Participants

Male and female volunteer and salaried firefighters from fire agencies across Australia were recruited for this study. Participants were screened and excluded from the study if they had been diagnosed with any form of heart disease, diabetes, respiratory or sleep disorders. Participants were then randomly assigned to either a control (CON) or sleep restriction (SR) condition. For purposes of analysis, participants in each condition were then matched for age, sex and body mass index (BMI). There were no differences with regards to age, firefighting experience and BMI between conditions (Table 1). Participants also completed pre- and post-testing health questionnaires to exclude any participants who became ill or sustained an injury directly prior to or during testing that could influence the inflammatory markers measured and confound any subsequent comparisons. As a result, a final sample of 18 firefighters in the CON condition and 17 firefighters in the SR condition completed this study (Table 1). Participation was voluntary and all participants gave written informed consent prior to commencing data collection. This study was approved by the Deakin University Human Research Ethics Committee.

**Table 1:** Characteristics of participants in each condition (mean ± standard deviation).

Characteristic	CON (*n* = 18)	SR (*n* = 17)
Age (years)	39 ± 16	39 ± 15
Male:Female (*n*)	15:3	15:2
Weight (kg)	85.1 ± 17.7	93.8 ± 20.2
Height (cm)	178.1 ± 7.7	177.8 ± 7.4
BMI (kg/m^2^)	26.8 ± 5.0	29.6 ± 5.5
Firefighting experience (years)	8.7 ± 9.3	10.2 ± 6.4

### Protocol

**Fig 1:**
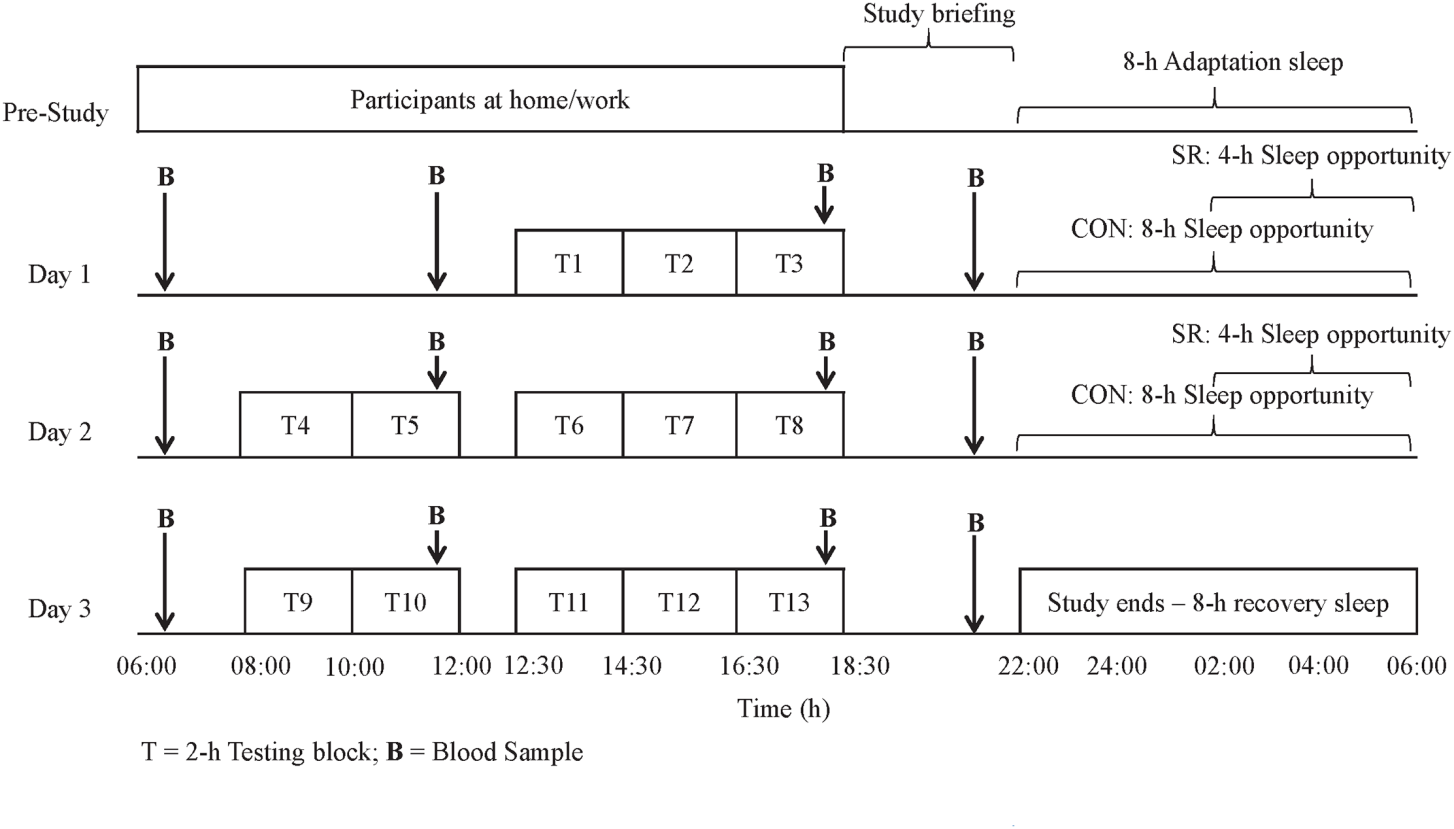
Firefighting work protocol for CON and SR conditions.

Participants in both conditions arrived at the testing venue and completed a familiarisation of all physical work tasks and physiological tests, followed by an adaptation night sleep (8-h sleep opportunity) in the testing environment before data collection began. All participants were then tested over a 3-day and 2-night simulated fire-ground deployment. On each of the 2 nights, participants in the CON condition had an 8-h sleep opportunity (i.e., 22:00–06:00; Fig 1). Conversely, participants in the SR condition had their bed time delayed, resulting in a 4-h sleep opportunity (i.e., 02:00–06:00) on each of the 2 nights (Fig 1). Participants in the SR condition were free to perform sedentary leisure activities (e.g., watching television, reading etc.) until the delayed bedtime. The duration of sleep restriction in this study was based on Australian wildland firefighters' self-reported average sleep per rest period on the fire-ground [1]. After completing the testing period, both conditions had an 8-h recovery sleep (in which no further measures were collected) to ensure, for the safety of all participants, that they were fully rested before leaving the testing venue and returning home (Fig 1). The testing environment was maintained at moderate temperatures (18–20°C) throughout the testing period in both conditions.

The timing of meals and the types of food and fluid available for consumption during testing were identical in both conditions and based on consultation with subject matter experts from Australian fire agencies. Adhering to fire-ground practices [35], food and drink intake during the study was *ad libitum* and the amount and type of food and drink ingested was recorded. This data was then extracted using the FoodWorks 7 nutrition software (2012 Xyris Software Pty Ltd, Australia). Although fluid consumption was recorded, the measurement of coffee and tea (i.e., caffeine) intake has only been reported in the results section. Energy and macronutrient intakes have also been described in the results section below.

### Experimental Procedures

Participants in both conditions were tested in small groups (of 3 to 5) over the 3-day wildland firefighting simulation. During the wake period, participants completed a 2-h testing block, 3 times on day 1 and 5 times on day 2 and 3 (Fig 1). Each 2-h testing block consisted of 55-min of simulated physical wildland firefighting work circuit, immediately followed by 20–25 min of physiological data collection (reported elsewhere [36]), 20–25 min of cognitive testing (outlined elsewhere [37]) and a 15–20 min rest period. Participants' cytokine levels were measured in both conditions at 4 identical time points across each of the 3 testing days (Fig 1).

#### Simulated physical firefighting work circuit

The physical work circuit comprised 6 simulated wildland firefighting tasks. Each of these tasks was designed to mimic the different physical demands involved in wildfire suppression [33]. Each have been recognised by incumbent firefighters and industry experts as being representative of repetitive movements and carry and drag movements that encompass key firefighting tasks frequently performed on the fire-ground [37]. The tasks included; lateral repositioning of a hose, rake-hoe work, hose rolling, charged hose advance, black out hose work, and static hold of a hose. These tasks were chosen because they were: 1) deemed to have the highest operational importance 2) the most physically demanding; and 3) the longest, most intense, or most frequently occurring tasks during wildfire suppression work [37, 38]. The tasks involved in the physical work circuit were completed in a pre-determined order with task work-to-rest ratios designed to mimic the performance of these tasks on the fire-ground [37, 38].

The performance of each physical task (i.e., repetitions completed for each task within each work period) was self-paced and therefore, a changing variable. Although performance of the physical tasks is not the focus of this study, it is possible the performance during the circuit (i.e., repetitions completed for each task) could interact with the cytokine responses and vice versa. Therefore, performance was recorded, but no significant differences in this component were demonstrated between conditions (findings reported elsewhere [36]), therefore it is unlikely physical performance had an impact on the cytokine response. Furthermore, the duration of the physical work circuit was the same for all participants, so it is also unlikely that this component had a confounding impact on the cytokine response.

#### Blood sampling and cytokine analysis

Participants provided fingertip capillary blood samples for the determination of IL-6, IL-8, IL-1β, TNF-α, IL-4 and IL-10 cytokine levels in blood plasma. Although previous emergency service-based studies have used venous blood samples when investigating cytokine levels [22–24], capillary blood samples were chosen because it is a minimally invasive method to conveniently obtain multiple daily blood samples from participants wearing personal protective clothing and performing repeated bouts of physical work. Some studies suggest that, due to a small local inflammatory response to the action of the pinprick, capillary blood samples can result in higher cytokine levels [39, 40]. However, recent evidence indicates a close correlation between venous and capillary plasma IL-6 responses during or post-exercise [39] and at rest [41]. Conversely, other reports have found that venous and capillary concentrations of TNF-α [40] and IL-6 [39] differed at rest. However, these studies [39, 40] did not control factors known to impact resting cytokine levels such as the time of day the sample was taken or whether or not the sample was taken under fasting conditions [42]. Control over these factors in the current study limits their confounding influence on cytokine levels at rest.

Participants samples were taken at 4 time points each day: a fasting baseline sample in the early morning (i.e., 06:15), late morning (i.e., 11:30), early evening (i.e., 18:15) and at night (i.e., 21:30; Fig 1). Prior to sample collection, participants held a heat pack in their hand to aid in blood flow to the fingertips. At every time point, a 500-μL sample of whole blood was taken from each participant in to a microtainer coated with K_2_ EDTA (Becton Dickinson ref: 365974). This process took between 1 and 10 minutes to complete and was the same in both conditions. Whole blood samples were centrifuged for 10 min at 5000 revolutions/min and the plasma was separated and stored frozen at ≤ -80°C.

The Milliplex Human MAP Cytokine immunoassay kit (Millipore, Billerica, MD) was used to profile the expression of inflammatory markers in the plasma samples of participants. The assay kits provide a mixture of microbead populations with distinct fluorescent intensities that are pre-coated with capture antibodies specific for each cytokine. The assay was performed according to the manufacturer’s instructions on the Bioplex 200 array reader (V.5.0, Bio-Rad Laboratories, Hercules, CA). The minimal detectable concentrations were 0.06 pg/mL, 0.42 pg/mL, 0.20 pg/mL, 0.05 pg/mL, 0.48 pg/mL and 0.07 pg/mL for IL-1β, IL-4, IL-6, IL-8, IL-10 and TNF-α, respectively. Cytokines intra- and inter-assay coefficients of variation were in acceptable ranges (Intra-assay 4.5–10.0%; Inter-assay 9.8–20.5%) for all analytes.

#### Sleep and activity monitoring

All participants had their sleep recorded on the adaptation night and the 2 study nights using standard polysomnographic (PSG) equipment (Compumedics E-Series, Australia). The adaptation night was designed to ensure participants’ sleep and cytokine responses during the study was not influenced by the lack of familiarity with the PSG equipment. Each night, PSG wire up and recording began at 21:00 for both conditions. From each sleep recording, participants' total sleep time (min) was calculated. In addition, participants wore activity monitors (*Actical* MiniMitter/Respironics, Bend, OR, USA) to measure sleep across the 2 nights leading in to the study. Participants’ physical activity during the simulation (2-h testing blocks) was also recorded through the use of activity monitors worn on the wrist. Physical activity data (captured in 1-min intervals) was downloaded using Actical software (version 3.10 MiniMitter/Respironics, Bend, OR, USA) and expressed as absolute counts. Further details regarding physical activity, including results, can be found in a previous study by Vincent et al. [36].

### Statistical Analyses

To decrease the biological variation associated with human plasma samples, outliers that had values greater than 2 standard deviations above the mean were excluded prior to the analysis [43]. Values that were below the detectable range of the Milliplex Human MAP Cytokine immunoassay kit were replaced with the minimal detectable concentration as advised in the protocol (Millipore, Billerica, MD). With the exception of TNF-α (for which raw values achieved normality and homogeneity of variance), all cytokines were natural log-transformed to achieve homogeneity of variance and normality of the residuals from the resulting mixed model analysis. The resulting diagnostic plots were visually examined and revealed no departures from the required assumptions. For ease of interpretation, cytokine values were back transformed to pg/mL in the figures presented.

Variables measured just once on an individual, or aggregated over occasions, (e.g., sleep duration, demographic characteristics and food and fluid intake) were analysed with the Analysis of Variance (ANOVA) method using GenStat software (GenStat *for Windows* 16.1 Edition. VSN International, Hemel Hempstead, UK). For repeated cytokine measurements, linear mixed models (LMM) were fitted by the restricted maximum likelihood (REML) method [44]. The LMM approach was used to investigate if participants’ individual profiles for each cytokine differed between conditions. This method also allows for the possibility of autocorrelation in the repeated cytokine measurements (i.e., samples and/or days) on each individual by including a model for the covariance structure. In addition, differences between the conditions in their linear trends and deviations from linearity were investigated via the incorporation of smoothing splines [45]. Model fit was assessed by Akaike Information Criterion (AIC) and small differences (ΔAIC) in this criterion compared to the minimum observed value in a set of candidate models were used to identify parsimonious models [46]. Predicted REML means constructed from the linear models fitted by the REML analysis were calculated and pairwise comparisons of these predicted means were used to examine differences in cytokines at each sample (or time point) and/or day.

The potential fixed effects investigated in the models fitted for each of the individual cytokine profiles were condition (CON or SR), day (day 1, day 2 or day 3) and time of day (06:15, 11:30, 18:15 and 21:30) along with potential interactions of condition by day, condition by time, day by time and condition by day by time. Random effects of group, profile (or participant), and a group by profile interaction were investigated by applying an independence (banded and unbanned), unstructured and power models for the within-subject autocorrelation. Finally, the REML procedure [44] was used to determine if a common spline was adequate to describe any non-linearity or if separate splines for each group or individual splines were required. Statistical significance was set at *p* < 0.05 and data are presented as means ± standard error of difference (SED) unless otherwise stated.

## Results

### Cytokines

**Fig 2:**
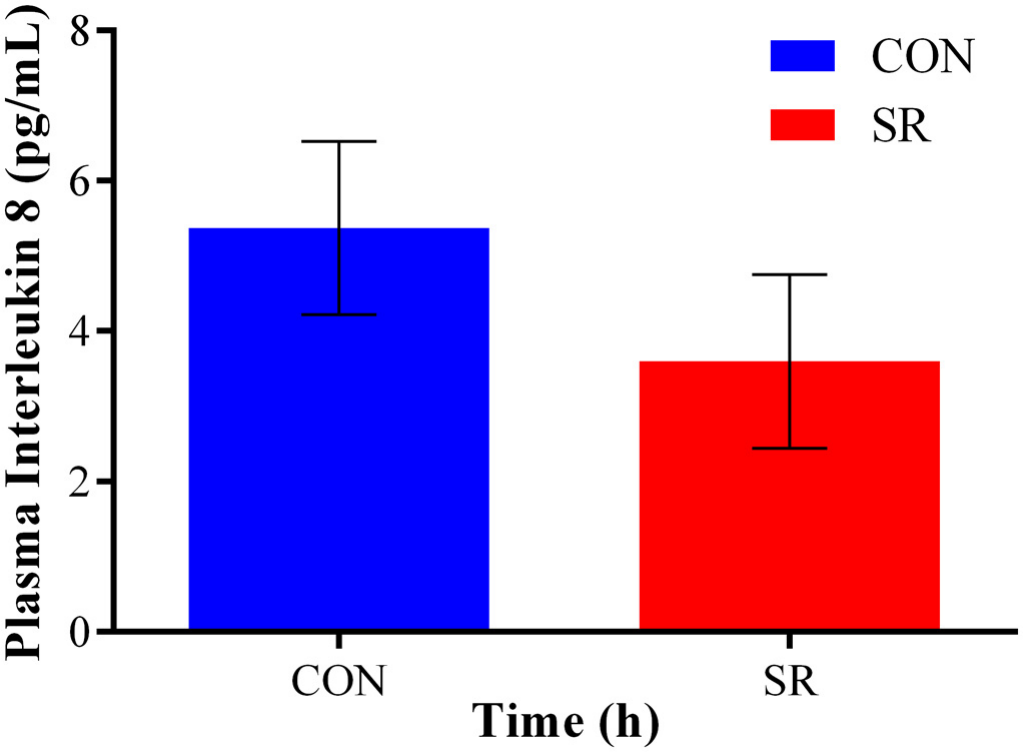
Predicted REML means for significant fixed effect of condition for IL-8 level in CON and SR conditions. Data was log-transformed prior to analysis. For ease of interpretation, values were back transformed to pg/mL.

**Fig 3:**
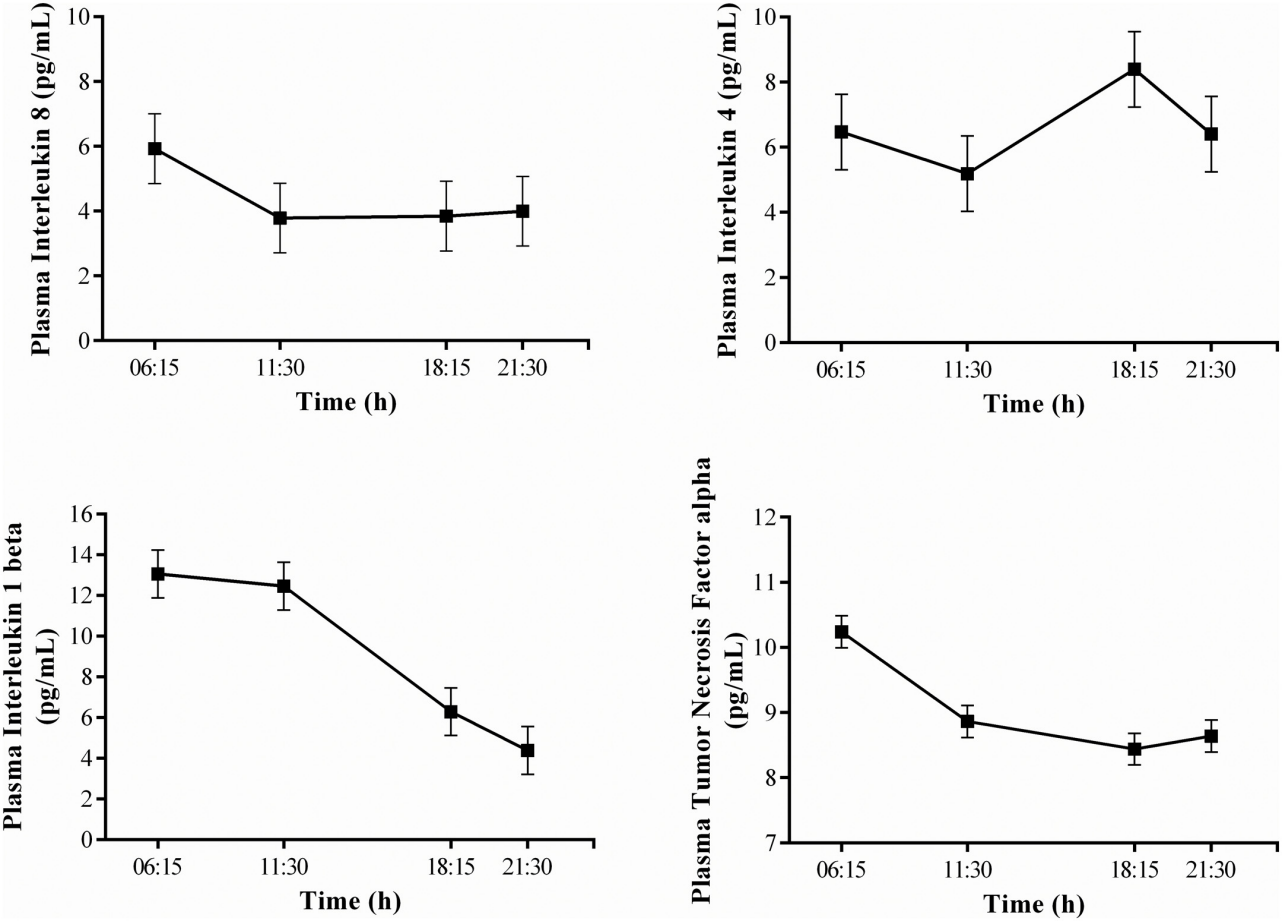
Predicted REML means for IL-8, IL-4, IL-1β and TNF-α across each daily time-point. Data was log-transformed prior to analysis. For ease of interpretation, values were back transformed to pg/mL.

Several LMM models were fit to the cytokine data, but for the majority of cytokines, the model with the lowest AIC and ΔAIC was the unstructured covariance model. Therefore, this model was considered to have the best fit to the data and selected as the final model for the analysis of each cytokine. The LMM demonstrated significant fixed effects for condition (*F* = 9.39, df = 1, *p* = 0.02) and time (*F* = 16.57, df = 3, *p* < 0.001) on log IL-8 levels, but no effect for day (*p* = 0.64). Predicted REML means for each condition showed that the CON condition had higher levels of log IL-8 (Fig 2), while predicted REML means at each time-point indicated that log IL-8 levels decreased across the day in both conditions (Fig 3).

**Fig 4:**
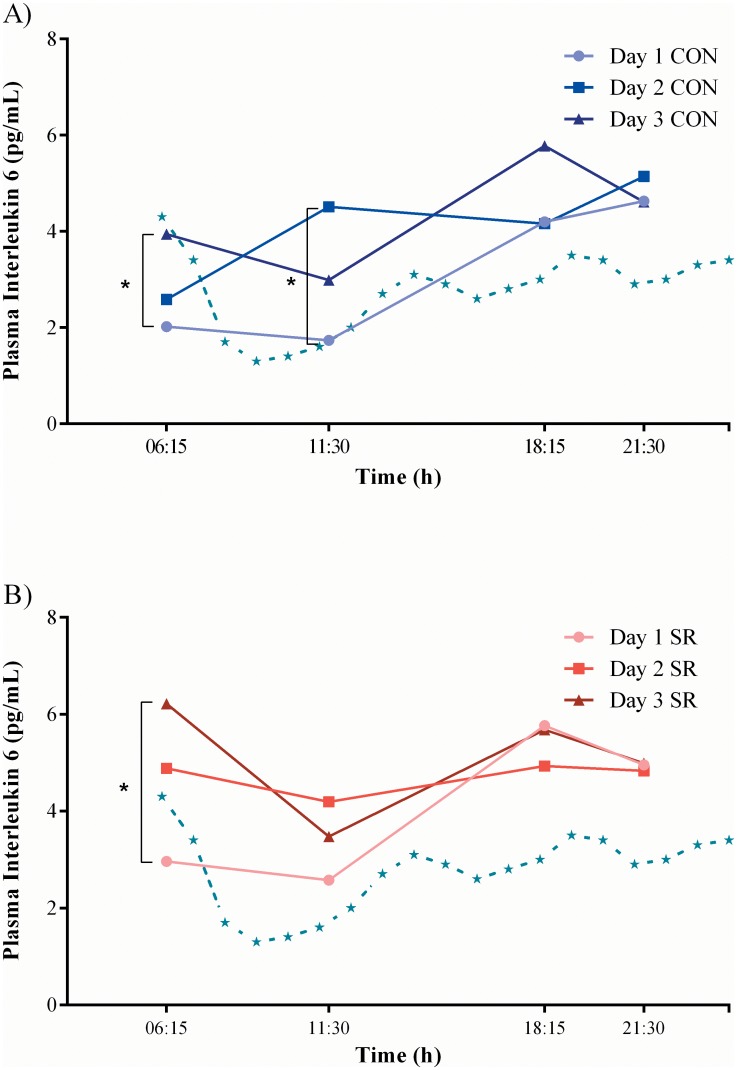
Predicted REML means for IL-6 profile across days in CON (A) and SR (B) conditions. Data was log-transformed prior to analysis. For ease of interpretation, values were back transformed to pg/mL and average standard error of difference = 1.29. NB: no shortened sleep prior to day 1. Dashed lines represent IL-6 levels for healthy young adults under control conditions (i.e, 8-h sleep opportunity) [47]. Significant differences between days within the same condition and time of day are indicated by * (*p* < 0.05).

The LMM demonstrated that there were significant fixed effects for time (*F* = 24.65, df = 3, *p* < 0.001) and day (*F* = 3.10, df = 2, *p* = 0.05) on log IL-6 levels. Predicted REML means indicated that log IL-6 levels in both conditions increased across time-points within a day and across each day. In addition, there were significant interaction effects for condition by time (*F* = 3.72, df = 3, *p* = 0.01) and day by time (*F* = 7.45, df = 6, *p* < 0.001) on log IL-6 levels. Pairwise comparison of predicted REML means further indicated that log IL-6 levels at 06:15 on day 3 were higher (+ 0.74) than at this time point on day 1 in both the SR (SED = 0.25, *p* = 0.03) and CON conditions (+ 0.67, SED = 0.24, *p* = 0.04; S1 Table; Fig 4). In addition, log IL-6 levels in the CON condition on day 2 at 11:30 (+ 0.95, SED = 0.27, *p* = 0.01) were higher than at this time point on day 1 in the CON condition (Fig 4). Further pairwise comparisons of predicted REML means indicated that there were no significant differences in log IL-6 levels between conditions at the baseline time point (i.e., day 1 at 06:15) or any of the other sampling time points. Therefore, it is likely the interaction is a reflection of different rates of change in log IL-6 levels between the SR and CON conditions, but not one that yields time point differences. Furthermore, significant fixed effects for time were found for log IL-1β (*F* = 21.79, df = 3, *p* < 0.001), raw TNF-α (*F* = 17.20, df = 3, *p* < 0.001) and log IL-4 levels (*F* = 3.65, df = 3, *p* = 0.02). For these fixed effects, predicted REML means demonstrated a decrease across time for log IL-1β and raw TNF-α levels, while predicted means for log IL-4 fell slightly from 06:15 to 11:30, followed by higher levels recorded in the evening and at night (i.e., 18:15 and 21:30; Fig 3). There were no further fixed or interaction effects demonstrated for the IL-1β, IL-4, TNF-α or IL-8 cytokine profiles, nor were there any significant effects for log IL-10 observed.

### Food and Drink

There was no difference in daily protein and fat intakes between conditions, but differences in carbohydrates (*p* = 0.01), energy (*p* = 0.02) and caffeine (*p* = 0.02) intake were demonstrated. Participants in the SR condition had a lower carbohydrate and energy (both *p* = 0.001) intake on day 3 compared to day 1, while both conditions had lower carbohydrate (SR *p* < 0.001; CON *p* = 0.01) and energy (SR *p* < 0.001; CON *p* = 0.001) intakes on day 3 compared to day 2. Furthermore, participants in the SR condition had lower carbohydrate (*p* = 0.01) and energy (*p* = 0.004) intakes on day 3 compared to this day for the CON condition. Compared to the CON condition, caffeine intake on day 1 and 2 was higher among participants in the SR condition (both *p* = 0.01), but intakes were not different to habitual caffeine consumption for this condition (*p* > 0.05).

### Sleep

Average total sleep time recorded for both the CON and SR conditions was not different on the adaptation night (*p* > 0.05; Table 2). The total sleep time on nights 2 and 3 was as expected given the sleep opportunity provided in each condition. Additionally, sleep duration measured across the 2 nights prior to the study using activity monitors was not significantly different to the adaptation night (i.e., 8-h sleep opportunity) or between conditions (both *p* > 0.05; Table 2) and therefore, participants were not sleep restricted before beginning the study.

**Table 2:** Total sleep time (mean ± SD) for each night in both conditions (h).

Night	CON	SR
Pre-study 1	7.3 ± 1.4	6.7 ± 0.9
Pre-study 2	6.7 ± 1.3	6.2 ± 1.4
1 (adaptation)	6.3 ± 0.9	6.4 ± 0.7
2	6.9 ± 0.4	3.6 ± 0.2*
3	6.9 ± 0.5	3.7 ± 0.2*

* = *p* < 0.001 between conditions

## Discussion

Higher IL-8 levels were recorded in firefighters who received an 8-h sleep each night when compared to the participants who had their sleep opportunity restricted to 4-h each night. Furthermore, there was an acute increase in IL-6 levels over successive days of firefighting work, irrespective of whether firefighters received an 8-h or 4-h sleep between days.

The higher levels of IL-8 in the 8-h sleep condition may be due to a higher level of physical activity than the sleep restricted condition. While there were no differences in the performance of physical firefighting tasks between conditions, Vincent et al. [36] found that compared to firefighters who had a restricted 4-h sleep, those who had an 8-h sleep displayed: 20.5% greater whole body physical activity (i.e., activity counts) during the rest periods between testing bouts (*p* < 0.001), 5.02% increase in physical activity during the firefighting work circuit (*p* < 0.05) and 11.92% increase across the 2-h testing block (*p* < 0.05). Vincent et al. [36] further reported that activity counts during both the physical work circuit (3.24%) and 2-h testing block (1.22%) increased over the course of the simulation among participants who had an 8-h sleep (both *p* < 0.05). Levels of IL-8 increase in response to long-duration physical activity with an eccentric contraction component [27]. A greater amount of physical activity in the CON condition during the rest periods and the work periods [36] may have resulted in elevated IL-8 levels.

The unaltered IL-8 levels in the SR condition are consistent with previous research [48, 49]. IL-8 is a bio-marker for cardiovascular health and accumulating evidence links chronically high levels of IL-8 with pathways that may increase the risk of CVD [20, 50]. The IL-8 levels observed among the current group of firefighters however, are below those more closely linked with CVD [20, 51] and there is currently no evidence of an increase in cardiovascular mortality [52] or a higher prevalence of CVD risk factors for Australian firefighters when compared to the general population [53]. However, in the United States of America (USA), cardiovascular events account for 35% of deaths among all firefighters (i.e., salaried, volunteer and wildland) [54]. Furthermore, an analysis of deaths among firefighters in the USA and estimates of time spent in firefighting duties reported that fire suppression activities were associated with an increased risk of death from coronary heart disease [55]. Therefore, examining repeated exposures to the acute physical demands involved in wildland firefighting and IL-8 release is warranted to investigate their potential role in the pathogenesis of cardiovascular-related health outcomes.

The increase in firefighters’ IL-6 levels across days was irrespective of the duration of sleep between shifts (4-h or 8-h opportunity). Sleep restricted to between 2 and 6-h per night over 5–10 nights, without physical work, has resulted in increased daytime levels of IL-6 [5, 7, 56], TNF-α [5, 6] and IL-1β [57]. Collectively, these findings suggest that longer periods of sleep restriction lasting five or more nights, may affect inflammatory activation, possibly via sleep loss-induced changes to vascular and metabolic function and disrupted sleep architecture [58, 59]. Given that firefighters can face extended wildfire deployments (e.g., >5 days) [60], future research should investigate the potential impact of prolonged periods of sleep restriction (i.e., >2 nights) on IL-6, TNF-α and IL-1β [5–7, 56, 57].

The 4-h sleep restriction period did not accentuate the rise in IL-6. Furthermore, the firefighters’ IL-6 levels in both conditions were high in comparison to young healthy adults who had an 8-h sleep opportunity and no physical activity (Fig 4) [47]. This may indicate that physical work was the major stressor in the current study. In contrast to our findings, Abedelmalek and colleagues [21] reported an increase in IL-6 following a 4-h sleep restriction period and four, 250-metre runs on a treadmill at 80% of personal maximal speed. In comparison to the self-paced work in our firefighting simulation, fixed speed work investigated by Abedelmalek et al. [21], although shorter, may have subjected participants to a higher intensity effort. Greater physical demands in combination with sleep restriction may explain why different IL-6 responses were observed between studies. Moreover, while similar durations of sleep restriction were investigated, Abedelmalek et al. [21] examined an early phase sleep opportunity (22:30–03:00). Wu et al. [61] found 4 nights of 3-h early phase sleep restriction caused a larger reduction in rapid-eye-movement (REM) sleep compared with later-night sleep restriction. Research in animal models indicates that a reduced amount of REM sleep had a positive association with increased IL-6 [62] and TNF-α [59]. Firefighting and other occupations (e.g., air crew, train and truck drivers) can require personnel to start work early in the morning, often causing truncated, early sleep phases [63]. Accordingly, future research needs to determine how early phase sleep restriction, and the associated changes in sleep architecture, impact the immune system of firefighters’ and workers in similar occupations completing physical work.

Morning IL-6 levels in both conditions increased from day 1 to day 3 of testing (Fig 4). This finding is in line with Gunderson and colleagues [23] who reported increased morning IL-6 levels among soldiers completing a 7-day training exercise with restricted sleep (1 h sleep per 24 h). However, the reported 11- and 7-fold increase in soldiers’ IL-6 levels from day 1 to day 2 and day 4 respectively [23], were higher than the 2-fold increase observed in the current study. The larger increase in soldiers’ IL-6 levels [23] could be explained by the long duration semi-continuous physical work [64] and extreme sleep restriction [34] they experienced, which are different to the intermittent physical work and partial sleep restriction typical for wildland firefighting [1] and simulated in the current study. While the observed changes in IL-6 (i.e., 1.9–2.5 pg/mL, Fig 4; S1 Table) are toward the low-end of systematic inflammation levels [28, 65], evidence suggests that similar levels of low-grade inflammation in IL-6, if chronic, are sufficient to increase the risk of adverse health outcomes [51, 66]. For instance, IL-6 >2.19 pg/mL was associated with an increased risk of all-cause mortality (cardiovascular diseases, cancers and other causes) among older adults across a 9-year period [51]. Findings from a 6-year study [66] showed that among healthy men at baseline, IL-6 levels were higher (1.81 pg/mL) in those who experienced a subsequent myocardial infarction. While firefighters in the USA appear to have a high risk of CHD-related deaths on duty [55], research in Australia is yet to investigate if firefighting demands are associated with cardiovascular events. Further research is needed to understand if wildland firefighters’ IL-6 levels are elevated chronically in response to repeated firefighting deployments across a fire season and over multiple fire seasons. Finally, IL-6 and IL-8 can induce white blood cell production [67] and it has been speculated that increased cytokines are responsible for heightened cell counts in cardiovascular disease and diabetes [68]. Therefore, it is important future longitudinal studies assess cytokine and blood cell differentials to determine how multiple immune responses interact and potentially impact on health.

Though the change in IL-6 levels was small, there was a significant increase in levels from day 1 to day 3 in both conditions at 06:15 and from day 1 to day 2 in the CON condition at 11:30 (Fig 4). While previous firefighting-based research found no change in IL-6 following short bouts of physical work [69], the findings from the current study support exercise and military literature demonstrating significant increases in IL-6 over successive days of both continuous [19] and intermittent [70] physical training. Physical work duration and intensity play a role in regulating IL-6 release [64]. Accumulating physical work output/performance across the three days may have contributed to the parallel rise in IL-6 levels in both conditions.

Low or depleted glycogen stores have been associated with an increased immune response following periods of long duration physical activity [71, 72], which could explain the increased IL-6 response on day 3 in both conditions. While intense periods of physical work can reduce appetite [73], the findings for the SR condition appear in contrast to reports that sleep loss may alter appetite regulation and lead to increased carbohydrate consumption [74]. However, the impact of sleep restriction on food intake has been largely investigated in the absence of physical work. In addition, previous energy restriction- and exercise-based studies that have specifically examined macronutrient intake and immune changes [71, 72] indicate that very low levels of carbohydrate (<10% of total energy intake from carbohydrates) during physical activity, with and without sleep loss, are necessary to elicit an acute immune response. In the current study, firefighters’ carbohydrate levels were higher in comparison to these studies (56% and 55% of total energy intake from carbohydrates on day 3 in SR and CON respectively) [71, 72] and remained within normal adult levels [75, 76]. It is therefore unlikely the lower carbohydrate intake among participants contributed to increased IL-6 levels on day 3. Further, while a high caffeine intake has been shown to increase IL-6 during physical activity (3 to 6 mg/kg body weight) [77], intakes in the current study were much lower (SR 2 mg/kg body weight, CON 1 mg/kg body weight).

Consistent with multi-day exercise training [19], firefighters’ IL-6 levels increased across the simulation. Lundeland et al. [24] and Gunderson et al. [23] reported that during a 7-day military training course with minimal sleep, IL-6 levels also increased to day 3 and day 4 respectively, but then decreased towards baseline. Following an increase in IL-6 across the first day of live wildfire suppression work, Main et al. [4] also found IL-6 levels to attenuate over the second day of work. In addition to pro-inflammatory activities, IL-6 has anti-inflammatory properties that lower other pro-inflammatory cytokines to return the immune system to homeostasis [12, 13]. For instance, data suggests that an increased IL-6 response during exercise can exert anti-inflammatory effects that inhibit TNF-α and IL-1β [25–27]. The increased levels of IL-6 among firefighters in the current study may have acted to suppress the release of TNF-α and IL-1β, resulting in declining levels of these cytokines across the day (Fig 3). Similar to IL-6, the IL-4 cytokine is immunomodulatory and therefore capable of exerting anti-inflammatory effects that inhibit IL-1β and TNF-α [11]. Therefore, increased IL-4 in the evening and at night may further explain the fall in TNF-α and IL-1β (Fig 3). Furthermore, compared to the inflammatory response typically related to exercise, severe inflammation (e.g., sepsis) has been associated with a distinctively different cascade of cytokines in the circulation (i.e., TNF-α, IL-1β and IL-6 in that order) [27, 28]. Therefore, increased levels of immunomodulatory cytokines IL-6 and IL-4 among firefighters in the current study, without an increase in TNF-α and IL-1β levels, could indicate a non-damaging response to the stress of the simulated physical firefighting.

The bidirectional feedback loop between cytokines and cortisol [29] may further explain the acute increase in IL-6 in the current study. Previously we demonstrated that 3 days of physical firefighting work separated by 2 nights of restricted sleep, resulted in an increased cortisol response over successive days of work [30]. These findings indicate that the body may be releasing more cortisol to compensate for the added stress of limited sleep while performing physical work. In turn, the increased release of cortisol may be exerting its anti-inflammatory effects [29, 31] and buffering the release of IL-6 in the SR condition. Given that cortisol may down-regulate proteins required for immune cell activation of IL-8 [32], higher cortisol levels previously reported [30] may result in greater inhibition of the IL-8 response among sleep restricted participants. To date, cortisol-induced anti-inflammatory effects for IL-8 have only been demonstrated in lipopolysaccharide (i.e., bacteria)-induced cytokine release [32]. Therefore, further research is needed to determine if cortisol has the same mediating impact on plasma IL-8 in response to physical work and sleep restriction. However, the potential for cortisol-induced suppression of IL-6 and possibly IL-8, suggests that the firefighters’ physiological stress-related changes were functioning effectively to maintain homeostasis of the immune system in response to simulated firefighting work with sleep restriction.

## Conclusion

This is the first wildland firefighting-based study to investigate the effect of sleep restriction and physical work on acute inflammatory stress responses. Findings demonstrate higher levels of IL-8 among participants who received an 8-h sleep each night when compared to those who had a restricted 4-h sleep. Participants’ IL-6 levels increased in both conditions suggesting that the sleep restriction duration and/or period may not have been a significant enough stressor to affect this cytokine over and above any disturbance caused by physical work. Considering the immunomodulatory properties of IL-6 and IL-4 [11, 25–27], the increase of these cytokines and the simultaneous decrease in TNF-α and IL-1β, could indicate a non-damaging response to the stress of simulated physical firefighting work. However, given the link between chronic low-level inflammation of IL-6 and IL-8, and several diseases such as atherosclerosis and Type 2 diabetes [66, 78], further research is needed to determine if wildland firefighters’ IL-6 and IL-8 levels are chronically elevated following repeated firefighting deployments across a fire season and over multiple fire seasons.

## Supporting Information

S1 Table Pairwise comparison of predicted REML means for logged IL-6 profile between days and within the same condition and time of day. (DOCX)
