# Supplementary material for: The Impact of Sleep Restriction and Simulated Physical Firefighting Work on Acute Inflammatory Stress Responses
Source: PLoS One. 2015 Sep 17;10(9):e0138128. doi: 10.1371/journal.pone.0138128 (PMC4574982; doi:10.1371/journal.pone.0138128)
Supplement: S1 Table — (DOCX) [file pone.0138128.s001.docx]

S1. Pairwise comparison of predicted REML means for logged IL-6 profile between days and within the same condition and time of day

| Condition | Time (h) | Comparison | Difference | LSD | *P* | Sig |
| --- | --- | --- | --- | --- | --- | --- |
| SR | 6.15 | Day 1 v Day 2 | 0.5016 | 0.6158 | 0.0927 | NS |
| SR | 11.30 | Day 1 v Day 2 | 0.4864 | 0.6927 | 0.1354 | NS |
| SR | 18.15 | Day 1 v Day 2 | 0.1573 | 0.5586 | 0.5136 | NS |
| SR | 21.30 | Day 1 v Day 2 | 0.0250 | 0.6415 | 0.9266 | NS |
| SR | 6.15 | Day 1 v Day 3 | 0.7423 | 0.6158 | 0.0259 | * |
| SR | 11.30 | Day 1 v Day 3 | 0.2983 | 0.6921 | 0.3294 | NS |
| SR | 18.15 | Day 1 v Day 3 | 0.0151 | 0.5545 | 0.9486 | NS |
| SR | 21.30 | Day 1 v Day 3 | 0.0071 | 0.6415 | 0.9791 | NS |
| SR | 6.15 | Day 2 v Day 3 | 0.2407 | 0.6214 | 0.3768 | NS |
| SR | 11.30 | Day 2 v Day 3 | 0.1880 | 0.6869 | 0.5250 | NS |
| SR | 18.15 | Day 2 v Day 3 | 0.1422 | 0.5586 | 0.5534 | NS |
| SR | 21.30 | Day 2 v Day 3 | 0.0321 | 0.6415 | 0.9058 | NS |
| CON | 6.15 | Day 1 v Day 2 | 0.2461 | 0.6037 | 0.3543 | NS |
| CON | 11.30 | Day 1 v Day 2 | 0.9543 | 0.6666 | 0.0131 | * |
| CON | 18.15 | Day 1 v Day 2 | 0.0093 | 0.5386 | 0.9673 | NS |
| CON | 21.30 | Day 1 v Day 2 | 0.1051 | 0.6230 | 0.6919 | NS |
| CON | 6.15 | Day 1 v Day 3 | 0.6677 | 0.6039 | 0.0355 | * |
| CON | 11.30 | Day 1 v Day 3 | 0.5432 | 0.6722 | 0.0948 | NS |
| CON | 18.15 | Day 1 v Day 3 | 0.3199 | 0.5385 | 0.1944 | NS |
| CON | 21.30 | Day 1 v Day 3 | 0.0035 | 0.6230 | 0.9893 | NS |
| CON | 6.15 | Day 2 v Day 3 | 0.4216 | 0.6037 | 0.1371 | NS |
| CON | 11.30 | Day 2 v Day 3 | 0.4111 | 0.6719 | 0.1833 | NS |
| CON | 18.15 | Day 2 v Day 3 | 0.3293 | 0.5386 | 0.1836 | NS |
| CON | 21.30 | Day 2 v Day 3 | 0.1086 | 0.6230 | 0.6822 | NS |
| LSD = least significant differences; NS = not significant * = *P* < 0.05 | | | | | | |
